# Proximity Labeling and Genetic Screening Reveal that DSG2 is a Counter Receptor of Siglec‐9 and Suppresses Macrophage Phagocytosis

**DOI:** 10.1002/advs.202406654

**Published:** 2025-01-15

**Authors:** Ying Wu, Yuyu You, Tingsong Jiang, Yuqi He, Qingchi Fan, Xinlei Zeng, Ting Li, Yuxing Lu, Liang Qi, Fengxia Zhou, Lingyu Sun, Danyang Wang, Yong Zou, Guigen Zhang, Yanqiu Yuan, Yang Mao

**Affiliations:** ^1^ School of Pharmaceutical Sciences Sun Yat‐sen University Guangzhou 510006 China; ^2^ State Key Laboratory of Oncology in South China Collaborative Innovation Center for Cancer Medicine Sun Yat‐sen University Cancer Center Guangzhou 510275 China; ^3^ Department of Blood Transfusion The Third Affiliated Hospital of Sun Yat‐sen University Guangzhou 510000 China; ^4^ Institute of Human Virology Department of Pathogen Biology and Biosecurity and Key Laboratory of Tropical Disease Control of Ministry of Education Zhongshan School of Medicine Sun Yat‐sen University Guangzhou 510080 China; ^5^ State Key Laboratory of Anti‐Infective Drug Discovery and Development School of Pharmaceutical Sciences Sun Yat‐sen University Guangzhou 510006 China; ^6^ Guangdong Provincial Key Laboratory of Drug Non‐Clinical Evaluation and Research Guangzhou 510006 China

**Keywords:** DSG2, genetic screening, phagocytosis, proximity labeling, siglec‐9

## Abstract

Cancer cells present sialylated glycoconjugates that modulate the activity of various immune cells within the tumor microenvironment through *trans* interaction with immunosuppressive Siglec receptors. Identifying counter receptors for Siglecs can provide valuable targets for cancer immunotherapy, but it presents significant challenges. Here, the identification of DSG2 (Desmoglein 2) as a dominant counter receptor of Siglec‐9 in melanoma cells is reported, using a workflow that combines the strength of proximity labeling and the advantage of CRISPR knockout screening. It is further demonstrated that the interaction between DSG2 and Siglec‐9 is mainly dependent on sialic acid‐bearing *N*‐glycans on DSG2. Importantly, blocking *trans* interaction between DSG2 and Siglec‐9 significantly enhances macrophage phagocytosis of melanoma cells and, to a lesser extent, other cancer cells. The work thus suggests sialylated DSG2 as a potential “don't eat me” signal molecule with therapeutic potentials in cancer immunotherapy.

## Graphical abstract

Identifying tumor‐specific counter receptors for Siglecs is challenging, but can provide valuable targets for immunotherapy. Here, proximity labeling combined with genetic screening to identify DSG2 as a dominant counter receptor of Siglec‐9 in melanoma. Blocking trans interaction between DSG2 and Siglec‐9 significantly enhances macrophage phagocytosis of melanoma cells, presenting sialylated DSG2 as a potential target in cancer immunotherapy.

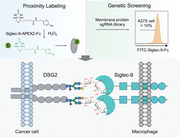

## 1 Introduction

Sialic acid‐binding immunoglobulin‐like lectins (Siglecs) are a family of lectin receptors mainly expressed on immune cells.^[^ ^1^, ^2^ ^]^ Most Siglecs contain immunoreceptor tyrosine‐based inhibitory motifs (ITIMs) in their cytoplasmic tails or associate with adapter proteins that have immunoreceptor tyrosine‐based activating motifs (ITAMs). By recognizing specific glycan ligands in *cis* or in *trans*, Siglecs play important roles in modulating immune cell functions.^[^ ^1^ ^–^ ^4^ ^]^

Siglec‐9 is a member of CD33‐related Siglecs, which has a predominant expression on myeloid cells.^[^ ^5^, ^6^ ^]^ Through its intracellular ITIM, Siglec‐9 transmits inhibitory signals into immune cells, which is exploited by various pathogens to dampen immune responses during infection.^[^ ^4^ ^]^ A well‐known example is group B Streptococcus (GBS), which suppresses the neutrophil oxidative burst and extracellular trap formation by displaying mimicry of host glycan ligands of Siglec‐9.^[^ ^7^ ^]^ Recent progress in immuno‐oncology indicates that cancer cells take advantage of Siglec‐9 signaling in tumor‐infiltrating immune cells by displaying specific glycan ligands on cell surfaces to evade immune surveillance.^[^ ^8^ ^]^ For example, engaging Siglec‐9 and its closely related Siglec‐7 allows a variety of tumors to modulate NK cell‐dependent tumor immunosurveillance^[^ ^9^ ^]^ and binding of a cancer‐specific MUC1 glycoform to Siglec‐9 induces tumor‐associated macrophage (TAM).^[^ ^10^, ^11^ ^]^ Furthermore, Siglec‐9 was reported to suppress phagocytosis in TAMs,^[^ ^12^ ^]^ ROS production in tumor‐associated neutrophils (TANs),^[^ ^13^ ^]^ as well as T cell activation in the tumor microenvironment.^[^ ^14^ ^]^ Therefore, identifying specific glycan ligands and counter receptors for Siglec‐9 in cancer cells could uncover potential “glyco‐immune checkpoints” and provide valuable targets for immunotherapy.^[^ ^15^ ^]^

However, the current knowledge about Siglec‐9 ligands is very limited due to the immense heterogeneity of glycans and numerous glycoconjugates that could serve as potential counter receptors of Siglec‐9. Based on glycan microarray analysis, Siglec‐9 preferably binds to terminal glycan structures containing sialic acid α2‐3‐linked to galactose.^[^ ^1^, ^16^, ^17^ ^]^ Nevertheless, the contribution of other structural moieties in glycans to Siglec‐9 binding remains undefined. For instance, an earlier study indicates that 6‐sulfation of sialyl‐Lewis x significantly increases binding affinity toward Siglec‐9,^[^ ^1^, ^17^ ^]^ which is supported by a recent NMR study showcasing the molecular features of Siglec‐9 binding^[^ ^18^ ^]^ and an array analysis using a library of sulfated terminal glycans.^[^ ^19^ ^]^ However, this notion is challenged by another study using a library of isomeric multiantennary *N*‐glycans, which shows that sulfation and fucosylation are not essential for Siglec‐9 binding.^[^ ^16^ ^]^ The discrepancies observed across different studies may suggest that different glycan scaffolds that present sialylated epitope affect its binding to Siglec‐9. Additionally, it remains a question whether Siglec‐9 differentially binds to *N*‐ or *O*‐glycans. The same study using a library of multi‐antennary *N*‐glycans shows that the binding to Siglec‐9 gets stronger as the number of antennas increases,^[^ ^16^ ^]^ indicating that avidity could be a contributing factor. However, clustered *O*‐glycans can provide such avidity for Siglec‐9 binding too, and cancer‐specific MUC1 glycoform has been shown to serve as a ligand for Siglec‐9 in the tumor microenvironment.^[^ ^10^ ^]^ A recent study using glyco‐engineered HEK293 cells for glycan display found that, at least in HEK293, the binding of Siglec‐9 depends primarily on *N*‐glycans.^[^ ^20^ ^]^ Therefore, different cells may display different sets of Siglec‐9 ligands and it remains to be determined what types of glycans serve as Siglec‐9 ligands in the tumor microenvironment and which glycoproteins act as Siglec‐9 counter receptors.

The difficulties in identifying specific Siglec‐9 ligands also lie in the weak affinity between Siglec‐9 and sialoglycans.^[^ ^21^ ^]^ The traditional ligand‐capturing approach based on affinity purification can hardly preserve sialic acids/Siglec‐9 interactions. In recent years, proximity labeling approaches have been used to identify the glycoprotein counter receptors for Siglecs and Galectins in live cells.^[^ ^22^, ^23^, ^24^, ^25^, ^26^, ^27^ ^]^ However, proximity labeling does not distinguish direct binding partners from bystanders, often generating a long list of candidates that can be difficult to functionally verify. Alternatively, CRISPR‐based genetic screenings have been successfully used to identify the glycan‐protein interactions between Siglec‐7 and CD43 in K562 cell line.^[^ ^15^ ^]^ However, previous genome‐wide CRISPR screening failed to identify specific ligands for Siglec‐9.^[^ ^15^ ^]^ Since CRISPR screenings enrich for genetic determinants of Siglec binding, it is possible that true ligands are confounded by genes that alter the expression or trafficking of membrane proteins.^[^ ^28^ ^]^ The potential redundancy of Siglec‐9 ligands expressed on cell surfaces may have further complicated the identification of true hits.^[^ ^15^ ^]^

In this study, we combined the strength of the proximity labeling approach to identify direct interactors and the advantage of a genetic screening with a focused library to find out functional determinants to profile Siglec‐9 ligands in cancer cells. We successfully discovered Desmoglein 2 (DSG2) as a functionally important counter receptor for Siglec‐9 in human melanoma cell line A375. Specific binding of *N*‐glycosylated DSG2 to Siglec‐9 contributed significantly to sialoglycan/Siglec‐9 mediated in *trans* “don't eat me” signal of A375 cells and suppressed phagocytosis. Importantly, the functional importance of DSG2/Siglec‐9 interaction could be replicated in lung cancer and cervical cancer cell lines with high DSG2 expressions, suggesting DSG2 might be a potential target in cancer immunotherapy.

## 2 Results

### 2.1 Establishment of a Proximity Labeling Strategy to Identify the Cellular Ligands of Siglec‐9

**Figure 1:**
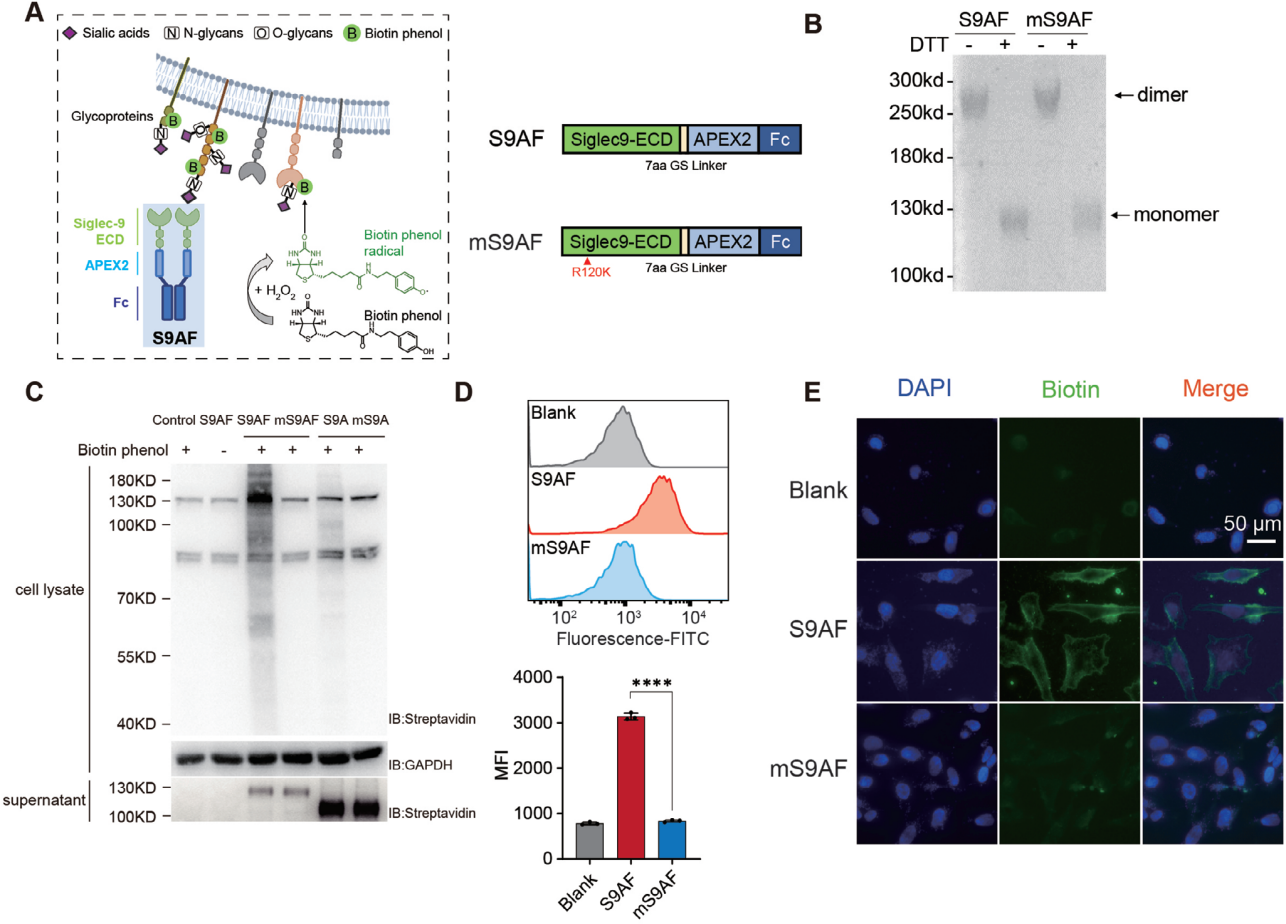
Establishment of a proximity labeling strategy for Siglec‐9 ligand identification. A) A schematic diagram showing in situ proximity labeling of cell surface ligands by the Siglec‐9 fusion protein. S9AF and mS9AF fusion proteins are represented on the right with the extracellular domain of either Siglec‐9 or its R120K mutant at the N terminal, followed by a GSGGGGS linker, the APEX2 enzyme, and the Fc fragment of human IgG1 at the C‐terminal. B) Non‐reducing SDS‐PAGE analysis of S9AF and mS9AF dimerization by Coomassie stain. C) Western blot analysis of cellular protein biotinylation by S9AF, mS9AF, S9A, and mS9A. No fusion proteins were added in the control. D) Flow cytometry analysis of A375 cell surface biotinylation using FITC‐streptavidin. Medium Fluorescence Intensity (MFI) of labeled cells were used for quantitative comparison. Cells not treated with any fusion proteins were used as a blank. Data are presented as mean values ± SEM (*n* = 3), and two‐tailed p‐values are calculated by unpaired Student's *t*‐test, ****p < 0.0001. E) Fluorescence microscopy images of A375 cells treated with S9AF or mS9AF. Cells not treated with any fusion proteins were used as a blank. Cells were stained with DAPI (blue) and Strep‐FITC (green). [Correction added on 23 January 2025, after first online publication: the oxygen atom was missing in the chemical structures in Figure 1 and TOC have an updated in this version.]

The Siglecs are known to cluster on live cell surfaces, suggesting that avidity is crucial for their signaling.^[^ ^4^, ^8^, ^29^ ^]^ To design a construct for proximity labeling that mimics the physiological state of Siglec‐9, we fused the extracellular domains of Siglec‐9 with APEX2, an engineered ascorbate peroxidase, and the Fc domain of human IgG1 to obtain a dimeric fusion protein (**Figure** **1**A). This fusion protein was named Siglec‐9‐APEX2‐Fc (S9AF). As a control, we generated a mutant protein, mSiglec‐9‐APEX2‐Fc (mS9AF), which lacks sialic acid binding capacity due to an introduced R120K mutation in Siglec‐9.^[^ ^6^ ^]^ Both fusion proteins were stably expressed in HEK293F cells and purified to homogeneity (Figure S1A, Supporting Information). The dimeric state of Siglec‐9 in both fusion proteins was confirmed through non‐reducing SDS‐PAGE (Figure 1B).

To evaluate the ability of S9AF to specifically label interactors of Siglec‐9 through the peroxidase activity of APEX2, we incubated purified S9AF with the human melanoma cell line A375, which was reported to express a high level of Sigelc‐9 ligands.^[^ ^9^ ^]^ After the addition of substrates for APEX2 including biotin‐phenol and H_2_O_2_ to the cells, biotinylated proteins in cell lysates were detected by western blot using the HRP‐conjugated streptavidin (Figure 1C). Using an optimized biotin‐phenol concentration and labeling time, we observed a dose‐dependent increase in protein biotinylation with increasing concentrations of S9AF (Figure S1B–D, Supporting Information). In contrast, mS9AF, the mutant protein incapable of binding to sialic acid, did not produce obvious signals over the background when used at the same concentration as S9AF (Figure 1C; Figure S1C,D, Supporting Information). This result demonstrated that S9AF was able to biotinylate cellular proteins depending on the recognition of sialic acids by Siglec‐9. Furthermore, the monomeric Siglec‐9 fusion protein (Siglec‐9‐APEX2, S9A) failed to produce significant signals at the same concentration used for S9AF (Figure 1C), supporting the notion that clustering is critical for the binding of Siglec‐9 to its sialylated ligands. To quantitatively analyze the avidity effect of Fc dimerization on ligand binding, we compared S9A with S9AF and observed a 10‐fold difference in the minimal amount of bait protein to achieve significant biotinylation in A375 (Figure S1E, Supporting Information). We also explored various options, including using *N‐* or *O‐*glycosylation inhibitors, sialidase treatment, and mS9AF, as a control in the proximity labeling experiment to reveal specific, glycan‐dependent interactors of Siglec‐9. As shown in Figure S1F,G (Supporting Information), sialidase treatment and mS9AF gave similar background labeling, while *N*‐ or *O*‐glycosylation inhibitor treatment resulted in different labeling patterns, which may represent different remaining glycan‐dependent interactions. To avoid any potential cellular damages caused by prior sialidase treatment, we chose mS9AF as our labeling control for the subsequent experiments.

Next, we measured the amount of labeled Siglec‐9 binding proteins by flow cytometry using FITC‐conjugated streptavidin. Labeling of A375 cells by S9AF, but not mS9AF, resulted in a significant increase in fluorescence (Figure 1D), confirming the specific labeling of Siglec‐9 binding proteins by S9AF. We also evaluated the S9AF‐mediated proximity labeling of cell surface ligands by fluorescence microscopy. As demonstrated in Figure 1E, notable fluorescence intensity was observed in cells treated with S9AF, but not in those treated with mS9AF. The fluorescence intensity was primarily localized on the cell surface. These findings thus suggested that sialic acid‐mediated interactions between Siglec‐9 and A375 cells were efficiently captured on the cell surface by S9AF. Consequently, the proximity labeling strategy using S9AF fusion protein holds promise for the identification of Siglec‐9 ligands.

### 2.2 Identification of Siglec‐9 Interactors by Quantitative Proteomics

**Figure 2:**
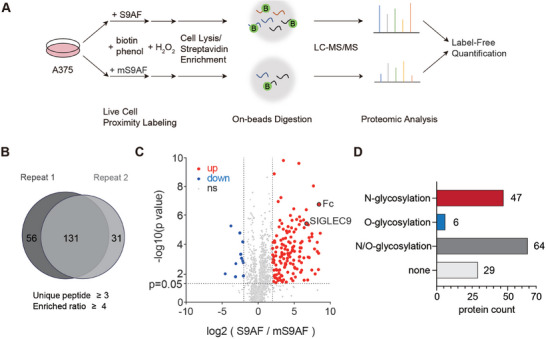
Label‐free quantitative proteomics for the identification of Siglec‐9 ligands in A375 cells. A) The workflow for the proximity labeling strategy integrated with label‐free quantitative mass spectrometry to identify Siglec‐9 ligands in A375 cells. B) Comparison of results from two biologically independent experiments showing an overlap of 131 protein ligands. All the protein ligands met the criteria of having three or more unique peptides identified and an enrichment ratio (S9AF/mS9AF) ≥ 4. C) The volcano plot illustrating significantly enriched Siglec‐9 ligands combining results from two biologically independent experiments (*p* < 0.05, ratio of S9AF/mS9AF ≥4). D) Known glycosylation types on Siglec‐9 ligands identified by proximity labeling according to UniProt and GlycoDomain Viewer.

To identify potential Siglec‐9 ligands on A375 cells, we adopted a quantitative proteomic approach by comparing the biotinylated proteome generated by the S9AF fusion protein to that by the inactive mS9AF protein (**Figure** **2**A). Following the treatment of either S9AF or mS9AF at a moderate concentration (12.5 ng µL^−1^), A375 cells were washed and lysed. Biotinylated proteins were captured on streptavidin magnetic beads and digested with trypsin on beads. The resulting digested peptides were then analyzed via LC‐MS and subjected to label‐free quantification. Using this workflow, we repeatedly enriched a total of 131 proteins (unique peptide ≥ 3, enrichment ratio ≥ 4) across two independent experiments (Figure 2B). Interestingly, Siglec‐9 itself was identified as a candidate interactor (Figure 2C), presumably because of self‐biotinylation by the homo‐dimer. Among the significantly enriched proteins (unique peptide ≥ 3, enriched ratio ≥ 4, *p* ≤ 0.05), 32% were known *N*‐glycosylated proteins, 4% were *O*‐glycosylated proteins, and 44% contained both types of glycosylations (Figure 2D). GO analysis revealed a high enrichment of plasma membrane or cell surface proteins and Biological Process analysis also found enrichment in proteins involved in cell‐cell/matrix adhesion (Figure S2, Supporting Information). These results were consistent with the function of Siglec‐9 lectin domain and suggested a preference for *N*‐glycan‐bearing counter receptors.

Although the APEX2‐mediated proximity labeling efficiently captured potential interacting proteins of Siglec‐9, it remained challenging to pinpoint functionally important ligands among hundreds of candidates. In addition, ≈20% of enriched proteins were not previously found to bear either *N*‐ or *O*‐glycosylations, thus could be bystander proteins labeled without direct interaction with S9AF. We therefore decided to take a CRISPR knockout‐based genomic screening approach to narrow down the candidate list as described below.

### 2.3 Identification of Functional Determinants of Siglec‐9 by CRISPR Knockout (KO) Screening

**Figure 3:**
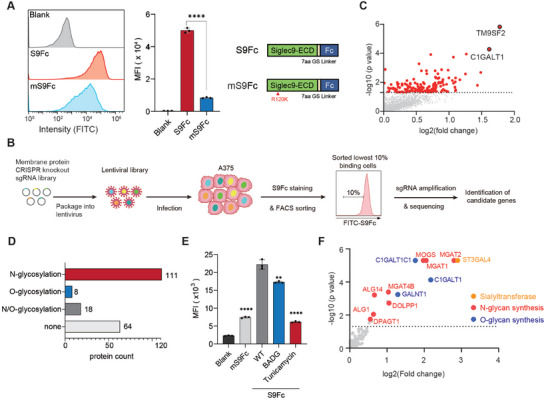
CRISPR knockout screening for Siglec‐9 ligand identification in A375 cells. A) Establishment of a flow cytometry‐based binding assay for Siglec‐9 and A375 cells. A375 cells were incubated with S9Fc or mS9Fc pre‐complexed to a FITC‐labeled anti‐hIgG antibody, analyzed on a flow cytometer (left), and quantified by MFI. Blank samples were incubated with FITC‐labeled anti‐hIgG antibody only. S9Fc and mS9Fc fusion proteins are represented on the right with the extracellular domain of either Siglec‐9 or its R120K mutant at the N terminal, followed by a GSGGGGS linker, and the Fc fragment of human IgG1 at the C‐terminal. B) The flowchart for the membrane protein CRISPR KO screening of potential Siglec‐9 ligands. A375 cells infected with the lentivirus membrane protein sgRNA library were incubated with S9Fc pre‐complexed with FITC‐labeled anti‐hIgG antibody and analyzed on a flow cytometer. Of the fluorescent cells, those exhibiting the lowest 10% fluorescence signals were collected and sent for sequencing analysis after sgRNA amplification. C) Dot plot of potential Siglec‐9 ligands identified through the CRISPR KO screening. Significantly enriched genes were shown in red (*p*‐value < 0.05). D) Known glycosylation types on Siglec‐9 ligands identified by CRISPR knockout screening according to UniProt and GlycoDomain Viewer. E) Flow cytometry analysis of Siglec‐9 binding to A375 cells in the presence of tunicamycin and BADG. F) Glycosyltransferase library CRISPR KO screening results. Genes involved in N‐glycan biosynthesis were shown in red, genes involved in O‐glycan biosynthesis were shown in blue, and genes encoding sialyltransferases were shown in orange. For (A,E), quantitative results are presented as mean values ± SEM (*n* = 3), and two‐tailed *p*‐values are calculated by unpaired Student's *t*‐test, *p < 0.05, **p < 0.01, ****p < 0.0001.

As an alternative approach, genetic screening holds promise for uncovering genes that are functionally involved in Siglec‐9 binding and signaling. To develop a CRISPR knockout‐based genetic screening method for Siglec‐9, we took advantage of the staining of A375 by recombinant Siglec‐9‐Fc (S9Fc) (**Figure** **3**A) and a focused single guide RNA (sgRNA) library featuring human membrane protein‐encoding genes (Figure 3B).^[^ ^30^ ^]^ Since localization of proteins to the cell surface is a prerequisite for them to serve as direct ligands of Siglec‐9, we anticipate that using a membrane protein‐focused library will help minimize the chance of enriching genes indirectly influencing Siglec‐9 binding. After stably expressing spCas9 in A375 cells, we infected cells with the sgRNA library containing 45901 sgRNAs. The infected cells were subsequently stained with S9Fc pre‐complexed with FITC‐conjugated anti‐human IgG. Cells with the lowest 10% fluorescence signals were then collected by fluorescence‐activated cell sorting (FACS). The sgRNA‐coding regions were amplified from the extracted genomic DNA of the collected cells and the unsorted control cells and sent for next‐generation sequencing (Figure 3B).^[^ ^31^ ^]^

Following data analysis, we observed a generally high false discovery (FDR) from the screening result. Wisnovsky et al. reported a genome‐wide CRISPR screen for ligands of Siglec‐7 and 9 and no specific glycoproteins were enriched for Siglec‐9.^[^ ^15^ ^]^ It was suggested that “Siglec‐9 ligands are expressed redundantly on a range of different underlying scaffolds.” We think that the generally high FDR seen in our screen may also reflect the redundancy of Siglec‐9 ligands on A375 cells, which causes limited fold change in binding upon knockout. To avoid missing significant hits, we opted to use a cutoff of *p* < 0.05 for hit identification. As a result, 200 genes were enriched (Figure 3C). Further analysis of the 200 genes revealed that 55.5% of them were reported to contain *N*‐glycans, 4% of them to contain *O*‐glycans, and 9% of them to contain both types of glycans (Figure 3D), which might be directly involved in the binding of S9Fc to A375 cells. However, 32% of the enriched genes were not previously reported to be glycoproteins (Figure 3D), which was a higher ratio than that we obtained from the proximity labeling, indicating the potential of the genetic screening to enrich genes indirectly affecting the binding of S9Fc. In line with this conclusion, the most significantly enriched gene in the screen was TM9SF2, a gene affecting the overall glycoprotein levels in cells.^[^ ^28^ ^]^

Interestingly, both proximity labeling and genetic screening enriched a higher proportion of proteins bearing *N*‐glycans than *O*‐glycans (Figures 2D and 3D), indicating a potential preference of Siglec‐9 for *N*‐glycans on cell surface proteins.^[^ ^20^, ^32^ ^]^ However, C1GALT1, the core 1 synthase for *O*‐glycans,^[^ ^33^, ^34^ ^]^ ranked the second in the CRISPR KO screening (Figure 3C). To have a better understanding of the contribution of these two glycosylation types, we treated A375 cells with tunicamycin (an inhibitor of *N*‐glycan biosynthesis) and benzyl 2‐acetamido‐2‐deoxy‐α‐D‐galactopyranoside (BADG, an inhibitor of *O*‐glycan biosynthesis), and evaluated the impact of the inhibitors on the binding of S9Fc to A375. As demonstrated in Figure 3E, tunicamycin almost abolished the interaction between S9Fc and A375 cells, while BADG reduced the binding of S9Fc by ≈30% (Figure 3E). To systematically demonstrate the structural determinants on glycans for Siglec‐9 binding we performed another CRISPR KO screening using a focused human glycosyltransferase gRNA library. In line with our previous findings, we found that the majority of the enriched genes were associated with the *N*‐glycosylation biosynthetic pathway, particularly in the MGAT family, including MGAT1, MGAT2, MGAT4B (Figure 3F). The MGAT family enzymes catalyze the transfer of a GlcNAc residue onto the mannose in the core structure of the nascent *N*‐linked glycan chain, giving rise to complex *N*‐glycan types (Figure S3, Supporting Information).^[^ ^35^ ^]^ It should be mentioned that another MGAT family member, MGAT3, which was reported to inhibit the extension of *N*‐glycans by generating the bisecting *N*‐Glycan,^[^ ^36^ ^]^ was not enriched in the screen. Notably, the core 1 synthase C1GALT1 was enriched again, together with its private chaperon C1GALT1C1 (COSMC) and the Polypeptide GalNAc Transferase 1 (GALNT1) (Figure 3F; Figure S3, Supporting Information), confirming the contribution of *O*‐glycosylation to the binding of Siglec‐9 to A375 cells. In addition, ST3GAL4, a α2,3 sialyltransferase modifying terminal galactose residues in both *N*‐ and *O*‐glycans, was identified as the most prominent hit in the screen (Figure 3F; Figure S3, Supporting Information), which was in line with the preference of Siglec‐9 for sialic acids α2‐3‐linked to galactose.^[^ ^1^, ^16^, ^17^, ^32^ ^]^ Together, these findings thus suggested that Siglec‐9 preferably recognizes the α2,3‐linked sialic acids in *N*‐glycans, however, the contribution of *O*‐glycans cannot be overlooked at least in A375 cells.

### 2.4 Identification of DSG2 as a Functionally Important Interactor of Siglec‐9

The fold change of potential interactors in proximity labeling reflects the binding specificities of Siglec‐9 to its ligands. However, it does not tell to what degree the potential interactor contributes to the overall display of Siglec‐9 ligands on A375 cells, thus does not predict if it is functionally important for Siglec‐9. On the other hand, CRISPR knockout‐based genetic screening reads out significant determinants of the overall display of Siglec‐9 ligands. Therefore, by comparing the list of potential Siglec‐9 ligands identified using the two aforementioned approaches, we are able to find functionally important interactors of Siglec‐9 on A375 cells.

**Figure 4:**
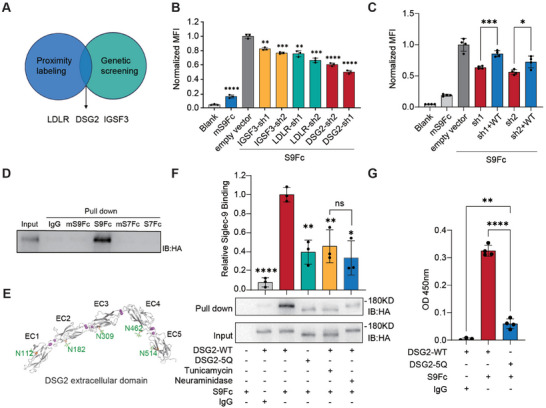
DSG2 in A375 cells specifically interacts with Siglec‐9. A) Both proximity labeling and CRISPR knockout screening identify LDLR, DSG2, and IGSF3 as Siglec‐9 ligands. B) Flow cytometry analysis of Siglec‐9 binding to A375 cells with genetic knockdown of DSG2, LDLR, or IGSF3 using shRNAs. A375 cells incubated with FITC‐labeled anti‐hIgG antibody only were used as blank and A375 cells infected with pLKO.1 empty vector was used as the control for comparison. C) Flow cytometry analysis of Siglec‐9 binding to A375 cells complemented with DSG2 upon shRNA knockdown. A375 cells infected with empty vectors were used as a control. D) Pull‐down of DSG2 overexpressed in HEK293T by Siglec‐9. E) A structural model illustrating the location of five *N*‐glycosylation sites on the extracellular domains of DSG2 (PDB: 5ERD). DSG2 was shown in a cartoon representation colored in light grey, calcium ions were shown as purple balls, and the first GlcNAc in the *N*‐glycan structure was shown in ball‐and‐stick representation colored in green. F) DSG2 binding to Siglec‐9 as analyzed by pull‐down experiments using S9Fc and HEK293T overexpressing full‐length DSG2 or DSG2‐5Q mutant or with Tunicamycin or neuraminidase treatment. Densitometry analysis of the western blot results was performed using ImageJ. G) Elisa analysis of DSG2 binding to Siglec‐9 using purified S9Fc and DSG2‐ECD or 5Q‐ECD. For (B,C,F,G) data are presented as mean values ± SEM (B and F, *n* = 3; C and G, *n* = 4), and two‐tailed *p*‐values are calculated by unpaired Student's *t*‐test, *p < 0.05, **p < 0.01, ***p < 0.001, ****p < 0.0001.

To our surprise, cross‐checking the functional significance of the proximity‐labeled interactors yielded only three proteins (IGSF3, LDLR, and DSG2) (**Figure** **4**A). Genetic knockdown of LDLR, IGSF3, or DSG2 by shRNAs resulted in a significant decrease of S9Fc binding to A375 cells, with DSG2 knockdown showing the most substantial reduction (Figure 4B; Figure S4A, Supporting Information). The exogenous expression of DSG2 partially restored the binding of Siglec‐9 to A375 (Figure 4C), which corresponded to the level of exogenous expression determined by the western blot (Figure S4B, Supporting Information). This result thus suggested that DSG2 could be a functionally important ligand for Siglec‐9. We next looked into the interaction between DSG2 and Siglec‐9 in detail.

To confirm the direct binding between DSG2 and Siglec‐9, we conducted a pull‐down experiment using purified S9Fc and HEK293T cells overexpressing DSG2. As shown in Figure 4D, full‐length DSG2 could be pulled down by S9Fc but not by the mutated mS9Fc, indicating a direct binding between Siglec‐9 and DSG2, and the interaction is dependent on sialic acid recognition. In addition, despite sharing 76% sequence identity with Siglec‐9, Siglec‐7 was unable to pull down DSG2 from the cell lysate (Figure 4D), suggesting that the specific interaction between DSG2 and Siglec‐9 can distinguish its closely related homolog.

Since we have demonstrated that Siglec‐9 preferably recognizes ligands bearing *N*‐glycans on cell surfaces, we next tested if *N*‐glycosylation on DSG2 plays a major role in mediating its binding to Siglec‐9. Five *N*‐glycosylation sites were annotated in UniProt in the extracellular domain of DSG2 (Figure 4E).^[^ ^37^, ^38^ ^]^ To evaluate if these glycosylation sites are crucial for Siglec‐9 binding, we engineered a full‐length DSG2 mutant with all five *N*‐glycosylation sites mutated to Gln (Q) (DSG2‐5Q). Overexpressing DSG2‐5Q in HEK293T cells resulted in a protein band with reduced molecular weight (Figure 4F). Similarly, treatment of HEK293T cells overexpressing wild‐type DSG2 with tunicamycin yielded the same downshift on the immunoblot (Figure 4F), confirming the presence of multiple *N*‐glycans in DSG2. Notably, removing *N*‐glycans from DSG2 by either approach diminished its interaction with Siglec‐9 to the level of neuraminidase treatment (Figure 4F; Figure S4C, Supporting Information). To further confirm the glycan‐dependent physical interaction between DSG2 and Siglec‐9, we next performed a direct ELISA experiment by immobilizing the purified extracellular domains of DSG2 (DSG2‐ECD) or DSG2‐5Q mutant (5Q‐ECD) (Figure S4D, Supporting Information) on a 96‐well plate and measuring binding to S9Fc using HRP‐conjugated anti‐IgG. As shown in Figure 4G, the wild‐type DSG2‐ECD exhibited much stronger binding to S9Fc compared to 5Q‐ECD.

Interestingly, both pull‐down experiment and ELISA showed that removing all five potential *N*‐glycosites did not completely disrupt the interaction between DSG2 and Siglec‐9, which suggested the possibility of a glycan‐independent interaction between these two molecules. Nevertheless, from these results, we can conclude that sialic acid‐bearing *N*‐glycans are the primary mediator in the recognition of Siglec‐9 and DSG2.

DSG2 is a calcium‐binding single‐pass transmembrane glycoprotein of the cadherin protein family known to mediate cell‐cell adhesion through dimerization via its extracellular domains.^[^ ^39^, ^40^ ^]^ There is increasing evidence indicating that DSG2 plays a crucial role in cancer cell proliferation and tumor progression.^[^ ^41^, ^42^, ^43^, ^44^ ^]^ In addition, DSG2 has been reported to be overexpressed in a large fraction of melanomas and be correlated with poor clinical outcomes. Given the specific interaction of DSG2 with Siglec‐9 in A375 cells, the function of DSG2 in melanoma could be related to Siglec‐9 and its immunosuppressive signaling in immune cells.

### 2.5 Disrupting *Trans* Interaction Between DSG2 and Siglec‐9 Enhances Phagocytosis of A375 Cells

The majority of human TAMs constitutively express Siglec‐9,^[^ ^45^ ^]^ and the signaling function of Siglec‐9 and Siglec‐E, the murine ortholog of Siglec‐9, was reported to suppress the phagocytosis of macrophage cells both in vitro and in vivo.^[^ ^12^, ^46^ ^]^ Therefore, we hypothesized that the *trans* interaction of DSG2 on cancer cells with Siglec‐9 on TAM might constitute a “don't eat me” signal and suppress phagocytosis.^[^ ^47^ ^]^

**Figure 5:**
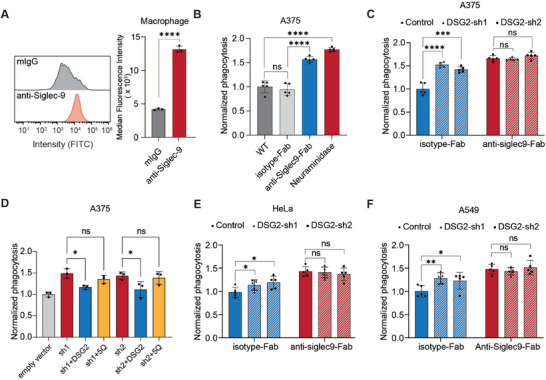
Blocking DSG2 and Siglec‐9 interaction enhances phagocytosis by macrophages isolated from PBMC. A) Macrophage cells induced by M‐CSF for 6 days express Siglec‐9 as measured by flow cytometry. Macrophage cells were incubated with anti‐Siglec‐9 mouse antibody or mouse IgG (mIgG) pre‐complexed to a FITC‐conjugated anti‐mIgG antibody and subjected to live cell flow cytometry analysis. A flow cytometry histogram showing Siglec‐9 expression levels. And expression level of Siglec‐9 on the macrophage surface was measured by the median fluorescence intensity (MFI) of stained macrophages. B) Phagocytosis of A375 cells by macrophages was enhanced by anti‐Siglec‐9 Fab or neuraminidase treatment. C) Phagocytosis of A375 cells by macrophages were enhanced by DSG2 knockdown, but not in A375 cells blocked with anti‐Siglec‐9 Fab. A375 cells infected with pLKO.1 empty vector was used as a control. D) Complementation of the DSG2 knockdown A375 cells with DGS2, but not DSG2‐5Q, reduced macrophage phagocytosis. A375 cells infected with empty vectors were used as a control. E,F) Phagocytosis of A549 and HeLa cells by macrophages were enhanced by DSG2 knockdown, but not in cells blocked with anti‐Siglec‐9 Fab. For (A–F) data are presented as mean values ± SEM (A and D, *n* = 3, B,C,E,F, *n* = 5), and two‐tailed *p*‐values are calculated by unpaired Student's *t*‐test, *p < 0.05, **p < 0.01, ***p < 0.001, ****p < 0.0001.

To test this hypothesis, we first confirmed that blocking Siglec‐9 signaling does enhance the phagocytosis of melanoma cells. In line with the previous report, human donor‐derived macrophages had a stable expression of Sigelc‐9 after differentiating from peripheral blood monocytes with macrophage colony‐stimulating factor (M‐CSF) (**Figure** **5**A; Figure S5A, Supporting Information).^[^ ^46^, ^48^ ^]^ Co‐culturing isolated macrophages with CFSE‐labeled A375 cells resulted in ≈32.2% phagocytosis, measured by flow cytometry (Figure S5B, Supporting Information). Blocking Siglec‐9 signaling with the Fab fragment of the anti‐Siglec‐9 antibody led to a 62% increase in phagocytosis (from 32.2% to 52.3% of the CFSE‐labeled A375 cells) (Figure 5B; Figure S5B top panels, Supporting Information). Additionally, removing sialic acids from A375 cells by neuraminidase treatment resulted in a similar increase in phagocytosis (from 32.2% to 57.8% of the CFSE‐labeled A375 cells) (Figure 5B; Figure S5B top panels and Figure S5C,D, Supporting Information). Together, these results indicated that macrophage‐expressed Siglec‐9 plays a suppressive role in phagocytosis of A375 through sialic acids‐mediated signaling. We therefore used this model to evaluate the role of DSG2 in the Siglec‐9 signaling.

Notably, knockdown of DSG2 alone resulted in a 46% increase of phagocytosis (from 34.9% to 51.2% of the CFSE‐labeled A375 cells) (Figure 5C left panel; Figure S5B bottom panels, Supporting Information). Since complete‐blocking Siglec‐9 signaling either with anti‐Siglec‐9 Fab treatment of macrophages or with neuraminidase treatment of A375 cells only raised the relative phagocytosis ratio by ≈60% (Figure 5B), this result indicated that DSG2 contributes significantly to the overall sialic acids‐mediated “don't eat me” signal from A375 cells. Importantly, knockdown of DSG2 did not cause an additional increase in phagocytosis when Siglec‐9 was blocked with anti‐Siglec‐9 Fab (Figure 5C right panel), confirming that the enhanced phagocytosis resulting from DSG2 knockdown was through its interaction with Siglec‐9.

The specificity of Siglec‐9 signaling by DSG2 was also confirmed by the complementation experiments, in which exogenous expression of wild‐type DSG2 in shRNA knockdown A375 resulted in a 22% reduction in macrophage phagocytosis as compared to the shRNA knockdown cells (Figure 5D; Figure S5E, Supporting Information). However, complementation with the DSG2‐5Q mutant did not have a statistically significant effect on phagocytosis.

To explore the potential role of the DSG2‐mediated “don't eat me” signal in other tumor types, we analyzed the expression profile of DSG2 in various tumor samples in TCGA and GTEx databases. We found a significant upregulation of DSG2 in patients with lung adenocarcinoma (LUAD), cervical squamous cell carcinoma (CESC), and pancreatic adenocarcinoma (PAAD) (Figure S5F, Supporting Information), which negatively associates with patients’ overall survival ratio (Figure S6A, Supporting Information). We therefore, chose three representative cell lines, A549, HeLa, and PANC‐1, for each cancer type and tested the contribution of DSG2 to the *trans* Siglec‐9 signaling in these cell models. Interestingly, knockdown of DSG2 only reduced the overall binding of A549 and HeLa to Siglec‐9 by 14.8%–26.7%, and did not significantly affect the binding of PANC‐1 to Siglec‐9 (Figure S6B–D, Supporting Information). In line with this result, although complete‐blocking Siglec‐9 signaling increased the phagocytosis of A549 by 47% and that of HeLa by 40%, knockdown of DSG2 in these cells only resulted in a modest increase in phagocytosis (Figure 5E,F, left panels). Because DSG2 knockdown in these cells did not further increase phagocytosis when macrophages were treated with anti‐Siglec‐9 Fab, the results suggested that DSG2‐induced phagocytosis is still through interacting with Siglec‐9 in these cells, albeit with a less significant effect (Figure 5E,F, right panels). These results suggest that in A549 and HeLa cells, there may be other sialic acids‐mediated “don't eat me” ligands. In addition, we noticed that α2‐3 linked sialylation was more abundant in A375 cells than other cell lines we tested (Figure S6E,F, Supporting Information). The sialyation level could be one of the reasons for the variability in the DGS2‐Siglec‐9 signaling axis across different cell types (Figure 5C,E,F).

## 3 Discussion

Cell surface sialic acids have been long perceived as a potential marker of “self‐associated molecular pattern (SAMP)” recognized by intrinsic inhibitory receptors in immune systems.^[^ ^49^ ^]^ In tumor microenvironment, cancer cells take advantage of this molecular pattern by engaging Siglecs on a variety of immune cells, constituting “glyco‐immune checkpoints”.^[^ ^15^ ^]^ However, this type of Siglec‐based glyco‐immune checkpoint receptor differs from more classical checkpoint receptors, such as PD‐1 and CTLA‐4, in lack of a defined counter receptor. Although targeted elimination of the entire tumor sialome has been utilized to enhance anticancer immune response,^[^ ^50^, ^51^, ^52^ ^]^ identifying a cancer‐specific dominant counter receptor for Siglecs would potentiate cancer immunotherapy in a more precise way.^[^ ^15^, ^53^ ^]^

Such counter receptor needs to have two attributes. First, it makes specific interactions with the dominant Siglec receptors in responsive immune cells. Second, it is upregulated in cancer cells so that it makes a significant contribution to *trans‐*Siglec signaling. Proximity labeling of interactors and genomic screening for binding determinants are two previously developed methods for identifications of potential counter receptors for Siglecs. Both methods have their advantages and disadvantages in enriching the two attributes of potential Siglec counter receptors. In this study, we have established a combined workflow by first using APEX2‐based proximity labeling to enrich direct interactors of Siglec‐9, followed by CRISPR knockout genetic screening to identify significant contributors to the binding of cell surface to Siglec‐9. Using this approach, we have discovered DSG2 both as a specific interactor of Siglec‐9 in A375 cells and a main contributor to the interaction between A375 and Siglec‐9, thus presenting as a potential counter receptor.

We have also demonstrated that the *trans* interaction between DSG2 on A375 melanoma cells and Siglec‐9 on macrophages has a significant impact on the Siglec‐9‐mediated immunosuppressive signaling and knocking down DSG2 releases immunosuppression and increases phagocytosis. The significant changes in phagocytosis upon DSG2 binding were reproducibly surprising. The anti‐phagocytotic effect of DSG2 was also observed for other cancer cell lines, such as A549 and HeLa, but not as pronounced as for A375 (Figure 5C,E,F). While DGS2 is likely not the only ligand for Siglec‐9 in A375 or other cancer cells we tested, it could be a major functional ligand in A375 cells, because its binding to Siglec‐9 might trigger strong suppressive signals for phagocytosis. Our findings thus suggest that DSG2 may function as a “don't eat me” signal in melanoma, warranting further investigation.

What differentiates DSG2 as a major ligand for Siglec‐9 in A375? Could it be a specific glycoform responsible for its interaction with Siglec‐9? We performed *N*‐glycan analysis on purified DSG2‐ECD and obtained *N*‐glycan profiles on the N112, N182, N309, and N462 sites of DSG2. We found that all four sites contained sialic acid‐bearing *N*‐glycans, but with varying occupancies (Figure S7A, Supporting Information). Interestingly, the most abundant glycan form was merely monosialylated biantennary *N*‐glycans (Figure S7B, Supporting Information). Several studies have indicated that avidity is crucial for Siglec‐9 signaling. In the case of DSG2, our data suggest that the avidity may arise from sialic acids on neighboring glycans, rather than from multiple antennaries at a single glycan. The formation of such avidity may be related to the structure of DGS2 and the distribution of glycosylation sites. Nevertheless, further investigation into the endogenous DSG2 glycan profile in A375 cells is required to fully explore the glycan features contributing to DSG2 binding to Siglec‐9 in the tumor environment. In addition, our pull‐down and ELISA also suggest that a glycan‐independent interaction may contribute to the specific recognition of DSG2 and Siglec‐9. As a matter of fact, DSG2 is a well‐known adhesion protein mediating cell‐cell interactions. By its adhesive nature, DSG2 binds to a variety of proteins, such as EGFR,^[^ ^54^, ^55^ ^]^ fiber knob protein^[^ ^56^ ^]^ and fatty acid binding protein 4.^[^ ^57^ ^]^ It has also been found that glycan‐independent interactions exist in some other glycan‐binding proteins (GBPs), including PSGL‐1^[^ ^58^ ^]^ and Siglec‐7.^[^ ^59^ ^]^ Our result of glycan‐independent interaction between DSG2 and Siglec‐9 is thus in line with these previous findings.

Importantly, knocking down DSG2 enhances the phagocytosis of different cancer cells to a different extent, indicating the contribution of DSG2 to the overall cell display of Siglec‐9 ligands is cell type‐specific. Different cancer cells might rely on different sialic‐acid‐presenting counter receptors and corresponding immunosuppressive Siglecs to evade immunosurveillance in the tumor microenvironment. Similar cases include the dominant role of CD43 in leukemia cell line K562^[^ ^15^ ^]^ and of CD24 in ovarian and breast cancer cells.^[^ ^47^ ^]^ The workflow presented in this work combined the strengths of proximity labeling and genetic screening and could provide a general approach to identify cancer cell‐specific counter receptors of Siglecs.

## Conflict of Interest

The authors declare no conflict of interest.

## Author Contributions

Y.W. and Y.You. contributed equally to this work. Y.Yuan. and Y.M. conceived the study and designed the experiments. Y.W. and Y.You. performed in vitro and cellular experiments. X.Z. performed data analysis for CRISPR screen. Q.F., T.J., and Y.L. performed the plasmid construction. F.Z., T.L., L.S. performed lentivirus production. D.W. and Y.H. performed cell culturing. Y.Z. and L.Q. collected the blood sample. G.Z. designed human membrane proteins CRISPR knockout library.

## Supporting information



Supporting Information

Supporting Information

## Data Availability

The data that support the findings of this study are available from the corresponding authors upon reasonable request. The mass spectrometry proteomics data have been deposited to the ProteomeXchange Consortium via the PRlDE partner repository with the dataset identifier PXD051557 [https://proteomecentral.proteomexchange.org/cgi/GetDataset?ID=PXD051557].
