# Supplementary material for: Proximity Labeling and Genetic Screening Reveal that DSG2 is a Counter Receptor of Siglec‐9 and Suppresses Macrophage Phagocytosis
Source: Adv Sci (Weinh). 2025 Jan 15;12(9):2406654. doi: 10.1002/advs.202406654 (PMC11884560; doi:10.1002/advs.202406654)
Supplement: Supplementary file 1 — Supporting Information [file ADVS-12-2406654-s001.docx]

Supporting Information

**Proximity Labeling and Genetic Screening Reveal that DSG2 is a Counter Receptor of Siglec-9 and Suppresses Macrophage Phagocytosis**

*Ying Wu^#^, Yuyu You^#^, Tingsong Jiang, Yuqi He, Qingchi Fan, Xinlei Zeng, Ting Li, Yuxing Lu, Liang Qi, Fengxia Zhou, Lingyu Sun, Danyang Wang, Yong Zou, Guigen Zhang, Yanqiu Yuan*, Yang Mao**

**Contents:**

1. Materials and Methods

2. Figures S1-S6

**1. Materials and Methods**

**Reagents**

Antibodies were purchased from Cell Signaling Technology (anti-HA cat# 2999, HRP-anti-rabbit cat# 7074, HRP-anti-mouse cat# 7076), Santa Cruz (GAPDH cat# sc-365062), Affinity Biosciences (DSG2 cat# DF3988), R&D system (Siglec-9 cat# MAB1139), Invitrogen (HRP-streptavidin cat# 434323, FITC-Goat-anti-Mouse-igG cat# A16079), Solarbio (FITC-streptavidin cat# SF068), Jackson (FITC-Goat-anti-human-igG cat# 109-095-088), Vector (Biotinylated-SNA cat# B-1305-2), and Biolegend (APC-anti-CD11b cat# 101211). Biotin-phenol, sodium azide, neuraminidase, iodoacetamide were from Sigma Aldrich. Human IgG, mouse IgG, tunicamycin, proteinase K, polybrene, protein A/G beads were from Beyotime Biotechnology. Streptavidin magnetic beads, CFDA-SE, protease inhibitors, blasticidin were from MedChemExpress.

**Cell culture**

Human HeLa (ATCC cat# CCL-2), HEK293T (Cellcook Biotech cat# CC4003), A375 (Cellcook Biotech cat# CC1801), PANC-1 (Cellcook Biotech cat# CC2401) were cultured in DMEM medium (Gibco). A549 (Cellcook Biotech cat# CC0202) were cultured in RPIM-1640 medium (Gibco). HEK293F (Thermo Fisher Scientific cat# R79007) were cultured in freestyle 293 medium (Gibco). The human peripheral blood mononuclear cells (PBMCs) were isolated from the peripheral blood of healthy donors (Guangzhou Blood Center) and cultured in RPIM-1640 medium (Gibco). The cell culture medium was supplemented with 10% FBS (Gibco), 100 U/mL of penicillin, and 0.1 mg/mL of streptomycin (Thermo Fisher Scientific). Cell lines were maintained at 37 ℃ under 5% CO_2_.

**Gene cloning and plasmid construction**

For expression of dimeric fusion protein S9AF, the DNA sequence encoding the extracellular domains of Siglec-9 (18-336) was amplified from a THP-1 cDNA library, APEX2 and the Fc fragment of hIgG1 coding sequences were codon-optimized and synthesized by Tsingke, and then fused together into pSec-Tag2A/myc-His through infusion reaction (HB-Infusion, HanBio). For expression of monomeric fusion protein S9A, the DNA sequences encoding the extracellular domains of Siglec-9 and APEX2 were fused into pCGS3. For expression of S9Fc, the DNA sequences encoding the extracellular domains of Siglec-9 and the Fc fragment of hIgG1 were inserted into pSec-Tag2A through infusion reaction. For expression of R120K mutant, site-directed mutagenesis was performed using Site-Directed Mutagenesis Kit (Beyotime) according to the manufacturer’s instructions.

For expression of DSG2, the full-length DSG2 coding sequence was PCR-amplified from an A375 cDNA library and cloned into pCDH-CMV-BSD through infusion reaction. The DSG2-5Q mutant was generated using Site-Directed Mutagenesis Kit.

For expression of DSG2-ECD, the extracellular domain of DSG2 coding sequence (50-609aa) was PCR-amplified from an A375 cDNA library and cloned into pSec-Tag2A through infusion reaction. The DSG2-5Q mutant was generated using Site-Directed Mutagenesis Kit.

For genetic knockdown, shRNA sequences of target genes were designed using GPP Web Portal (<https://portals.broadinstitute.org/gpp/public/>) and synthesized by Tsingke. The annealed oligos were then cloned into PLKO.1 using T4 ligase (NEB). The shRNA sequences are listed in Table S1, Supporting Information.

The DNA sequences of constructed plasmids were confirmed by Sanger Sequencing. The primers for plasmid construction are listed in Table S2, Supporting Information.

**Proteins expression and purification**

The S9AF, mS9AF, S9A, mS9A, Siglec-9-Fc, mSiglec-9-Fc, DSG2-ECD and DSG2-ECD-5Q fusion proteins were expressed in HEK293F by transient transfection of corresponding expression plasmids using PEI (Polysciences) following manufacture’s instruction. After 4 days of culturing, the medium were collected and filtered through a 0.45 µm filter. The protein was purified by protein A/G beads or Ni-NTA Beads (TransGen Biotech) according to the manufacture’s instruction, then confirmed and quantified by SDS-PAGE. The purified proteins were maintained in Tris buffer (pH 7.4) containing 150mM NaCl and stored at -80°C for future usage.

**Proximity labeling and detection**

For live cell proximity labeling, A375 cells were plated in 10 cm dishes at a density of 1×10^7^ cells per plate. The next day, cells were gently washed with PBS and incubated with 12.5 µg/mL S9AF or mS9AF and 500 μM biotin-phenol in DMEM for 30 mins at 37 °C. After incubation, H_2_O_2_ was added to a final concentration of 1 mM, and the plate was gently shaken for 1 min at room temperature. After aspirating the medium, labeling reaction was stopped with the quencher solution containing 5 mM Trolox, 10 mM sodium ascorbate and 10 mM sodium azide in PBS. For *N*- or *O*-glycosylation inhibition, A375 cells were treated with 0.25 μg/mL Tunicamycin for 24 h or 0.5 μM BADG (Macklin) for 72 h before analysis.

For western blot analysis, cells were washed twice with PBS and lysed with RIPA buffer (Genstar) containing protease inhibitor cocktail. Protein concentrations were measured by BCA assay and normalized for sample loading. Cell lysates were separated on 10% SDS-PAGE and immunoblotting was performed following standard Tris-glycine-SDS protocols. Biotin labeling was detected by HRP-conjugated streptavidin. All antibodies used in the western blot are listed in Table S3, Supporting Information.

For flow cytometry analysis, A375 cells were collected after treated with 2mM EDTA and stained with FITC-streptavidin for 30 min at 4℃. Cells were then analyzed on a CytoFLEX(Beckman). Medium Fluorescence Intensity (MFI) of labeled cells was used for quantitative comparison.

For fluorescence imaging, A375 cells were fixed with 4% paraformaldehyde at room temperature for 30 min and washed with PBS. The cells were subsequently incubated with FITC-conjugated streptavidin (SF068, Solarbio,1:200) in PBST (pH 7.4) for 2 h at room temperature and washed three times with PBS. Imaging was performed on a fluorescence microscope (Zeiss, AXIO Imager A1) and ZEN Blue Lite 2.3 software was used for image capture and data analysis.

**Sample preparation for proteomics**

Proximity labeling was performed as described above. After labeling, A375 cells were washed once with PBS and resuspended in lysis buffer (PBS, 0.1% SDS, 0.1% Trition X-100, 1 mM PMSF, 1 x protease inhibitor cocktail). Cell lysates were sonicated and cleared by high-speed centrifugation. The protein concentrations were measured by BCA assay and normalized before enrichment. For enrichment of biotin-labeled proteins, each sample was incubated with pre-washed streptavidin magnetic beads (50 μL slurry) for 2 h at room temperature on a rotator. After incubation, the streptavidin beads were sequentially washed with 0.5 mL of 2 M urea in PBS once and 0.5 mL of 50 mM ammonium bicarbonate three times. The beads were then resuspended in 100 μL 50 mM ammonium bicarbonate. Proteins were reduced with 10 mM DTT for 45 min at 60 °C and then alkylated with 20 mM iodoacetamide for 30 min at room temperature in the dark. Sequencing grade trypsin (Promega) was added to a final concentration of 10 ng/μL and the mixture was incubated with shaking at 800 rpm at 37 °C overnight. The supernatant was adjusted to pH 2~3 using trifluoroacetic formic acid (Thermo Fisher), desalted on reverse-phase C18 StageTips (Empore, Sigma) and eluted with 0.1% formic acid (Thermo Fisher) in 50% methanol. The eluted peptides were dried via vacuum centrifugation.

**LC-MS/MS analysis**

Peptide samples were dissolved in 0.1% formic acid for MS analysis. Samples were analyzed using an EASY-nLC 1200 system coupled to a Q Exactive Plus mass spectrometer equipped with a Nanospray-electrospray source (Thermo Fisher Scientific). For liquid chromatography, mobile phase A consists of water with 0.1% FA and mobile phase B consists 80% acetonitrile, 0.1% FA, and 19.9% water. Nano-LC was operated on a single analytical column. Samples were injected onto a C18 column (C18-AQ, Dr.maisch, 1.9 μm, inner diameter 75 μm, length 20 cm) and eluted in a 120 min gradient (95 min of 3% to 32% solvent B, 10 min of 32% to 100% solvent B, 15 min of 100% solvent B) at a flow rate of 200 nL/min. The mass spectrometer was set to acquire full-scan MS spectra (355–1700 m/z) for a maximum injection time of 100 ms at a mass resolution of 70,000 and an automated gain control (AGC) target value of 5e^5^. The dynamic exclusion was set to 60s with an exclusion window of 10 ppm. In HCD scans, the collision energy was set at 27 in fixed collision energy mode. The AGC target was set to 5e^4^, and maximum injection time was 75 ms. All MS/MS spectra were acquired in the Orbitrap with a resolution at 35,000 in profile mode.

**Proteomics data analysis**

MS data processing for all raw files was performed using Proteome Discoverer (PD) version 2.4 software (Thermo Fisher Scientific). All raw files were searched with Sequest HT against the proteome database of homo sapiens (202310UP000005640, 82685). The precursor mass tolerance was set to 10 ppm and fragment ion mass tolerance to 0.02 Da. Trypsin was set as the specific proteolytic enzyme, and a maximum of two missed cleavages was allowed. Carbamidomethylation (C, +57.02146) was specified as static modifications. Oxidation (M, +15.994915) was specified as a dynamic modification. The target false discovery rate (FDR) of 1% was set for proteins and peptides. Proteins in the inclusion list met the criteria of having at least three unique peptides identified.

Label-free quantification was used for differential proteomic between S9AF- or mS9AF-treated samples. LFQ ratios obtained from Proteome Discoverer were transformed with log2 (x), and P values were obtained using one-way ANOVA test with three technical replicates per sample.

Candidates were selected based on an MS1 intensity ratio ≥4 in the S9AF-treated sample as compared to the mS9AF-treated sample, and P < 0.05, with the requirement of at least three unique identified peptides. Results from the quantitative analysis are listed in Table S4, Supporting Information. The mass spectrometry proteomic data have been deposited to the ProteomeXchange Consortium via the PRIDE^[1]^ partner repository with the dataset identifier PXD051557.

**Flow cytometry-based Siglec-9 binding assay**

0.4 μg S9F or mS9F and 0.8 μg FITC-conjugated goat anti-human IgG were pre-complexed by incubating in 100 µL FACS buffer (PBS plus 0.5% BSA) on ice for 30 min and then incubated with 4 × 10^5^ target cells for 30 min. The samples were always kept in the dark and on ice. After being washed twice with FACS buffer, the samples were analyzed on a CytoFLEX S (Backman). The gating strategy for live cells was set up based on FSC/SSC, and single cells was set up on FSC-A/FSC-H in gated live cells, then analyzed in FITC channel. Collected data were further processed using FlowJo (v10.0.7) software. Siglec-9 binding was quantified as normalized mean fluorescence intensity (MFI) in the FITC channel. For analysis of *N*-glycosylation contribution in Siglec-9 binding, A375 were treated with 0.25 μg/mL Tunicamycin for 24 h before Siglec-9 binding assay. As for *O*-glycosylation, A375 cells were treated with 0.5 μM BADG (Macklin) for 72 h. At least three independently performed experiments were measured for each sample.

**Lentiviral production and transduction**

The CRISPR library targeting 4,978 genes encoding human membrane proteins was a gift from Zhang Guigen’s Lab. It contains 45,901 sgRNAs, among which 512 were non-targeting sgRNAs as negative controls. For lentiviral packaging, HEK293T cells were plated onto twenty 100 mm dishes at a concentration of 3 × 10^6^ cells per dish in completed medium. Cell medium was changed to serum-free DMEM at 70% confluence. A mixture of 7 µg of sgRNA library plasmid and 7 µg lentiviral packaging plasmids (5 µg psPAX2, 2 µg VSVG) in 1 mL serum-free DMEM was prepared for each plate. 42 µL transfection reagent PEI was then added to the plasmid mixture solution and vortex. After incubation at room temperature for 20 minutes, the transfection mixture was added to each 100 mm plate dropwise as the media on the plate was swirled constantly. After additional 4 h incubation at 37°C in 5% CO_2_, the medium was changed to complete medium. Virus production was allowed to proceed for 48 h. Lentiviral media was then collected from each plate, centrifuged at 2000 rpm for 5 minutes to clear any cellular debris, aliquoted to 15 mL centrifuge tubes and stored at -80 °C until the day of transduction.

For library virus transduction, A375 stably expressing spCas9 (A375-Cas9 cells) was obtained by transducing lentivirus of spCas9 expression vector (Addgene #52962) and selecting by blasticidin. Multiplicity of infection (MOI) was measured by determining the titer of lentiviral media that produced 30% cell viability following infection and 2 µg/mL of puromycin selection for 48 h. 2 x106 A375-Cas9 cells were seeded in twenty-five 100 mm dishes. 24h later, 8 µg/mL polybrene and the volume of lentiviral media previously determined to give an MOI of 0.3 were added to the dishes. After 24 h, media was refreshed with 2 µg/mL puromycin, and cells were maintained under antibiotic selection for 48 h. An uninfected control plate treated with 2 µg/mL puromycin had cellular viability less than 1%. After this point, media was changed to complete media to allow gene editing for an additional 5 days.

The glycoGene CRISPR library^[2]^ includes 4 high potency sgRNA against 227 gene targets related to *N*-glycan, *O*-glycan, Glycosaminoglycan biosynthesis. 25 sgRNAs were non-targeting sgRNAs as negative controls. The sgRNA sequences were synthesized by GENEWIZ and cloned into Lenticrispr V2. The lentiviral production and transduction were described as above.

**CRISPR KO screening**

After transduction and antibiotic selection, 4 x10^7^ cells were collected as unsorted control, flash frozen and stored at -80 °C. For cell sorting, the 4 x10^7^ cells were staining according to the method described in FACS analysis of Siglec-9 binding, and then resuspended in 2 mL FACS buffer. Cells were then passed through a cell strainer to remove any aggregates. Cells were sorted on a Beckman CytoFLEX SRT. The analysis gate for live cells was set up based on FSC/SSC, and the gate for single cells was set up on FSC-A/FSC-H of gated live cells. The collection gate was defined as the lowest 10% population of total FITC-stained cells. Sorted cells were immediately pelleted.

Genomic DNAs from unsorted control and sorted cells were isolated as previously described. Briefly, cells were lysed with lysis buffer (50 mM Tris, 50 mM EDTA, 1% SDS, pH 8), and incubated with protease K (ST535, Beyotime) at 55℃ overnight followed by Rnase A (2158, Takara) treatment at 37℃ for half an h. After proteins removal by adding 7.5 M ammonium acetate and centrifugation at 12,000 rcf for 10 min, genome DNA was precipitated with isopropanol. After washing with 70% ethanol and air drying, genome DNA was dissolved in nuclease-free H2O and the concentration was determined using a Nanodrop spectrophotometer. The sgRNA-coding sequences were amplified by a two-step PCR reaction^[3]^ and subsequently analyzed on an Illumina NextSeq. FASTQ sequence alignment to the library file was performed using MAGeCK^[4]^. The sgRNA counts of the sorted sample were compared to that of the unsorted control sample. Genes with a positive p-value less than 0.05 are considered as significantly enriched candidate genes. The full results are listed in Table S5, Supporting Information.

**Real-time qPCR**

Gene knockdown was achieved by lentiviral transduction of shRNA expression plasmids. The shRNA lentiviral production and transduction were performed as above. To determine knockdown efficiency, total RNAs were extracted using RNAiso Plus kit (TaKaRa) and cDNAs were prepared from 1 μg RNA using HiScript Ⅲ RT SuperMix (Vazyme) following the manufacturer’s instruction. Real-time qPCR was performed using SYBR qPCR Master Mix (Vazyme) in a CFX Connect Real-Time PCR System (BioRad). Relative gene expression was calculated based on the delta-delta Ct method using ACTB as an internal control. The sequences for all qPCR primers are listed in Table S6, Supporting Information.

**Neuraminidase treatment**

To remove cell surface sialic acids, cells were treated with 10 mU neuraminidase per million cells in 100 μL PBS for 1 h at 37°C. Western blot analysis was performed using the biotinylated sambucus nigra lectin to evaluate cell surface sialic acids.

**Immunoprecipitation**

HEK293T expressing DSG2-WT or DSG2-5Q were grown to 70% confluent in six-well plates. Cells were washed with cold PBS and lysed in ice-cold lysis buffer (50 mM Tris-HCl, pH 7.5, 150 mM NaCl, 5 mM MgCl_2_, 0.5% TritonX-100, 1 mM EDTA and protease inhibitor cocktail). After centrifugation at 21,000g for 10 minutes, the supernatants were collected and protein concentration were determined by BCA assay. Samples were diluted in lysis buffer to a final concentration of 2 mg/mL. Meanwhile, 3 μg Siglec-9-Fc fusion protein was pre-complexed with 30 μL protein A magnetic beads (GenSript) in 150 μL lysis buffer by rotating at room temperature for 30 min. Cell lysates were then added to Siglec-9-coated beads and rotated at 4 °C for 4 h. Then, beads were washed three times with lysis buffer without protease inhibitors. The immunoprecipitated proteins on beads were eluted by heating in 1x SDS loading buffer with 10 mM DTT at 98°C for 10 minutes followed by immunoblotting analysis of the DSG2 proteins.

**ELISA**

The purified 50 ng of DSG2-WT or DSG2-5Q were coated in 96-well plates using 10 mM sodium carbonate buffer (pH 9.6) overnight at 4 °C and washed three times with PBST. The proteins were subsequently incubated with 100 ng S9AF or hu-IgG in PLI-P Buffer (pH 7.4) for 1 h at room temperature and washed three times with PBST. The proteins were then incubated with HRP conjugated anti-hu-IgG antibody (1:5000) in PLI-P Buffer for 1 h at room temperature and washed three times with PBST. The absorbance at 450 nm was used for quantitative comparison.

**DSG2 *N*-glycosylation type analysis**

For *N*-glycosylation analysis of DSG2, 0.5 μg of purified DSG2-ECD was resuspended in 100 μL 50 mM ammonium bicarbonate. Proteins were reduced with 10 mM DTT for 45 min at 60 °C and then alkylated with 20 mM iodoacetamide for 30 min at room temperature in the dark. Sequencing grade chymotrypsin (Roche cat# 11418467001) was added to a final concentration of 10 ng/μL and incubated at 25 °C overnight. The supernatant was adjusted to pH 2~3 using trifluoroacetic formic acid (Thermo Fisher), desalted on reverse-phase C18 StageTips (Empore, Sigma), and eluted with 0.1% formic acid (Thermo Fisher) in 50% methanol. The eluted peptides were then dried via vacuum centrifugation. LC-MS/MS analysis was perform as previously described.

MS data processing of raw files was performed using Glyco-Decipher (version 1.0.4) software^[5]^. The raw files were searched against the protein sequence of homo-DSG2 (Q14126). The MS1 mass tolerance was set to 5 ppm and the MS2 mass tolerance was set to 20 ppm. Chymotrypsin was set as the specific proteolytic enzyme, and a maximum of three missed cleavages was allowed. Carbamidomethylation (C, +57.02146) was specified as static modifications. Oxidation (M, +15.994915) was specified as a dynamic modification. The target false discovery rate (FDR) of 1% was set for proteins and peptides.

**Phagocytosis assay**

To generate macrophages, PBMCs were purified from peripheral blood through density gradient centrifugation using Ficoll (Biosharp) according to the manufacturer’s instruction. Then, monocytes were isolated from the PBMCs by adherence and differentiated into macrophages by stimulating with 50 ng/mL M-CSF (Sino Biological) for 6 days.

Phagocytosis assay was performed by co-culturing tumor cells and donor-derived macrophages. Prior to co-culturing, cancer cells were stained with 1 μM CFSE. Then, 2 × 10^5^ cancer cells were co-cultured with macrophages at a ratio of 5:1 in 96-well round bottom plates with or without 10 μg/mL anti-Siglec-9 (clone 191240, R&D) Fab fragments for 2h. After co-culturing, cells were collected by digestion with 2mM EDTA and staining with APC-labeled anti-CD11b for 30 min at 4℃. Cells were then analyzed on a CytoFLEX S (Beckman). The analysis gate for live cells was set up based on FSC/SSC, and the gate for single cells was set up on FSC-A/FSC-H of gated live cells. Macrophages were gated as APC positive population. Phagocytosis was measured as the number of CD11b^+^FITC^+^ macrophages, quantified as a percentage of the total CD11b^+^ macrophages. We repeated at least three independently stained and analyzed samples per group.

**Statistical analysis**

Biochemical experiments were routinely repeated for at least three times. Statistical analysis was performed and *P* values were calculated using a two-tailed unpaired Student *t* test unless otherwise specified.

**Safety statement**

No unexpected or unusually high safety hazards were encountered.

**References**

[1]. Perez-Riverol, Y., J. Bai, C. Bandla, D. Garcia-Seisdedos, S. Hewapathirana, S. Kamatchinathan, D.J. Kundu, A. Prakash, A. Frericks-Zipper, M. Eisenacher, M. Walzer, S. Wang, A. Brazma, and J.A. Vizcaino, *The PRIDE database resources in 2022: a hub for mass spectrometry-based proteomics evidences* [J]*.* Nucleic Acids Research, 2022. **50**(D1): p. D543-D552.

[2]. Narimatsu, Y., H.J. Joshi, Z. Yang, C. Gomes, Y.-H. Chen, F.C. Lorenzetti, S. Furukawa, K.T. Schjoldager, L. Hansen, H. Clausen, E.P. Bennett, and H.H. Wandall, *A validated gRNA library for CRISPR/Cas9 targeting of the human glycosyltransferase genome* [J]*.* Glycobiology, 2018. **28**(5): p. 295-305.

[3]. Canver, M.C., M. Haeussler, D.E. Bauer, S.H. Orkin, N.E. Sanjana, O. Shalem, G.-C. Yuan, F. Zhang, J.-P. Concordet, and L. Pinello, *Integrated design, execution, and analysis of arrayed and pooled CRISPR genome-editing experiments* [J]*.* Nature Protocols, 2018. **13**(5): p. 946-986.

[4]. Li, W., H. Xu, T. Xiao, L. Cong, M.I. Love, F. Zhang, R.A. Irizarry, J.S. Liu, M. Brown, and X.S. Liu, *MAGeCK enables robust identification of essential genes from genome-scale CRISPR/Cas9 knockout screens* [J]*.* Genome Biology, 2014. **15**(12).

[5]. Fang, Z., H. Qin, J. Mao, Z. Wang, N. Zhang, Y. Wang, L. Liu, Y. Nie, M. Dong, and M. Ye, *Glyco-Decipher enables glycan database-independent peptide matching and in-depth characterization of site-specific N-glycosylation* [J]*.* Nature Communications, 2022. **13**(1).

**2. Figure S1-S6**


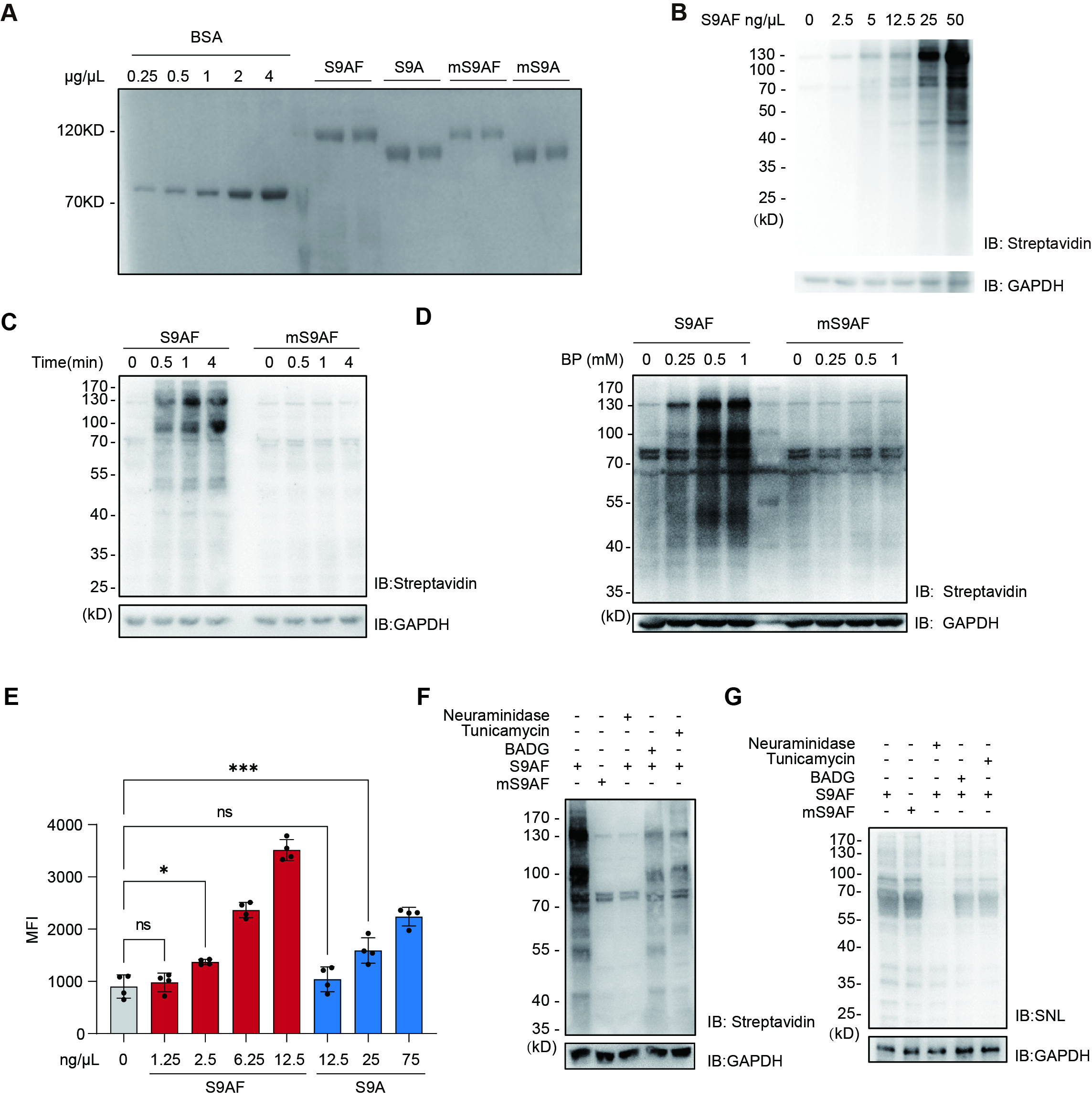


**Figure S1. A.** Purity confirmation and quantification of purified S9AF/mS9AF and S9A/mS9A by reducing SDS-PAGE using Coomassie stain. **B.** Western blot analysis of biotinylated proteins after proximity labeling with increasing concentrations of S9AF. **C.** Western blot analysis of biotinylated proteins after proximity labeling with increasing labeling time **D.** Western blot analysis of biotinylated proteins after proximity labeling with increasing concentrations of biotin-phenol **E.** Flow cytometry analysis of A375 cell surface biotinylation, after proximity labeling with increasing concentrations of S9AF and S9A, using FITC-streptavidin (right). Medium Fluorescence Intensity (MFI) of labeled cells was used for quantitative comparison. Data are presented as mean values ± SEM (n = 4), and two-tailed *P*-values are calculated by unpaired Student's *t* test, **P* < 0.05, ****P* < 0.001. **F.** Western blot analysis of cellular protein biotinylation by S9AF, mS9AF, with or without the treatment of BADG, tunicamycin, neuraminidase. **G.** Western blot analysis of cell surface sialic acids in A375 cells with BADG or tunicamycin or neuraminidase treatment using SNL.


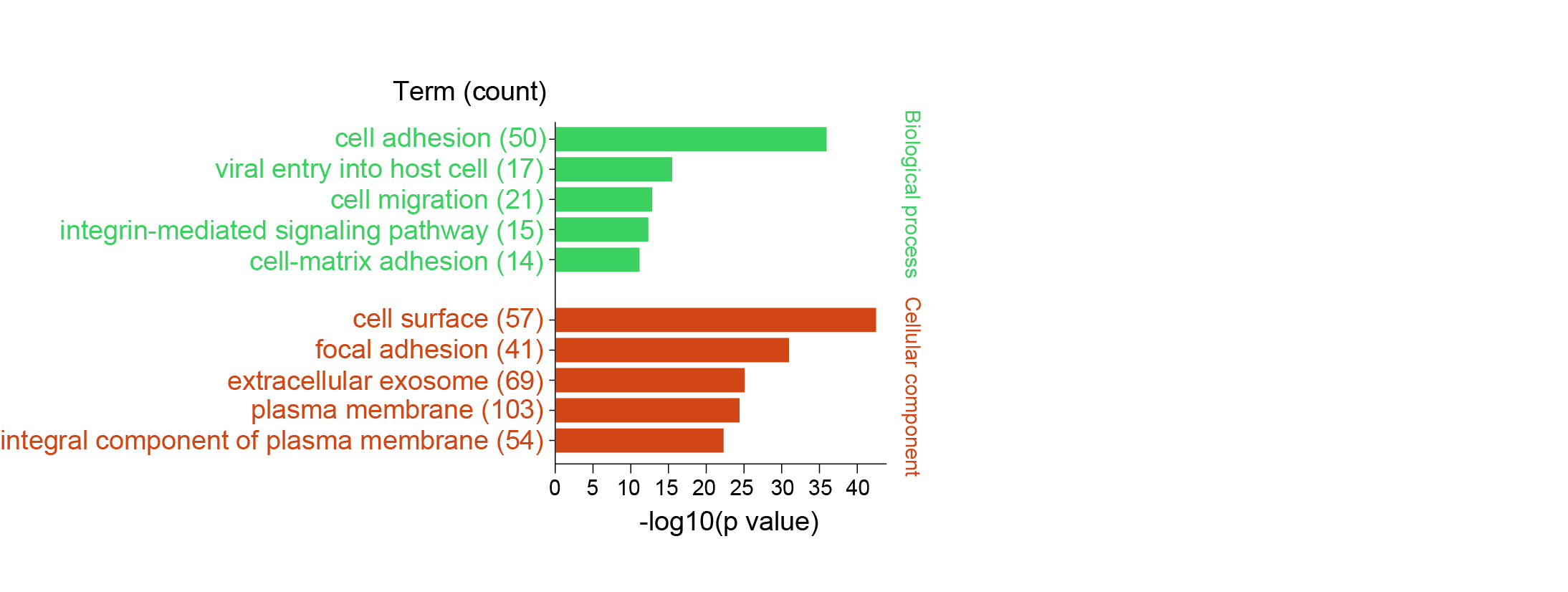


**Figure S2.** GO analysis shows that significantly enriched proteins are predominantly localized to the cell surface and associated with cell adhesion.


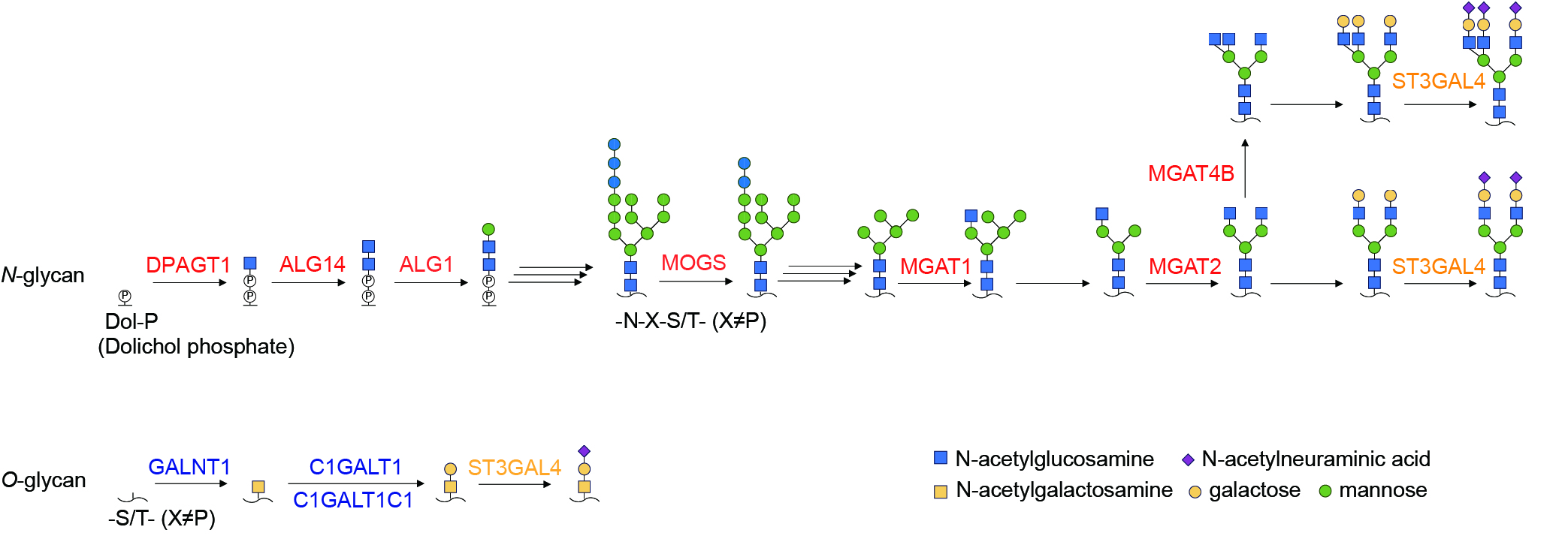


**Figure S3.** Genes enriched in CRISPR KO screening with human glycosyltransferase sgRNA library are shown in their corresponding glycan biosynthesis pathways. Genes involved in *N-*glycan biosynthesis are shown in red, *O-*glycan biosynthesis in blue and those encoding sialyltransferases in orange.


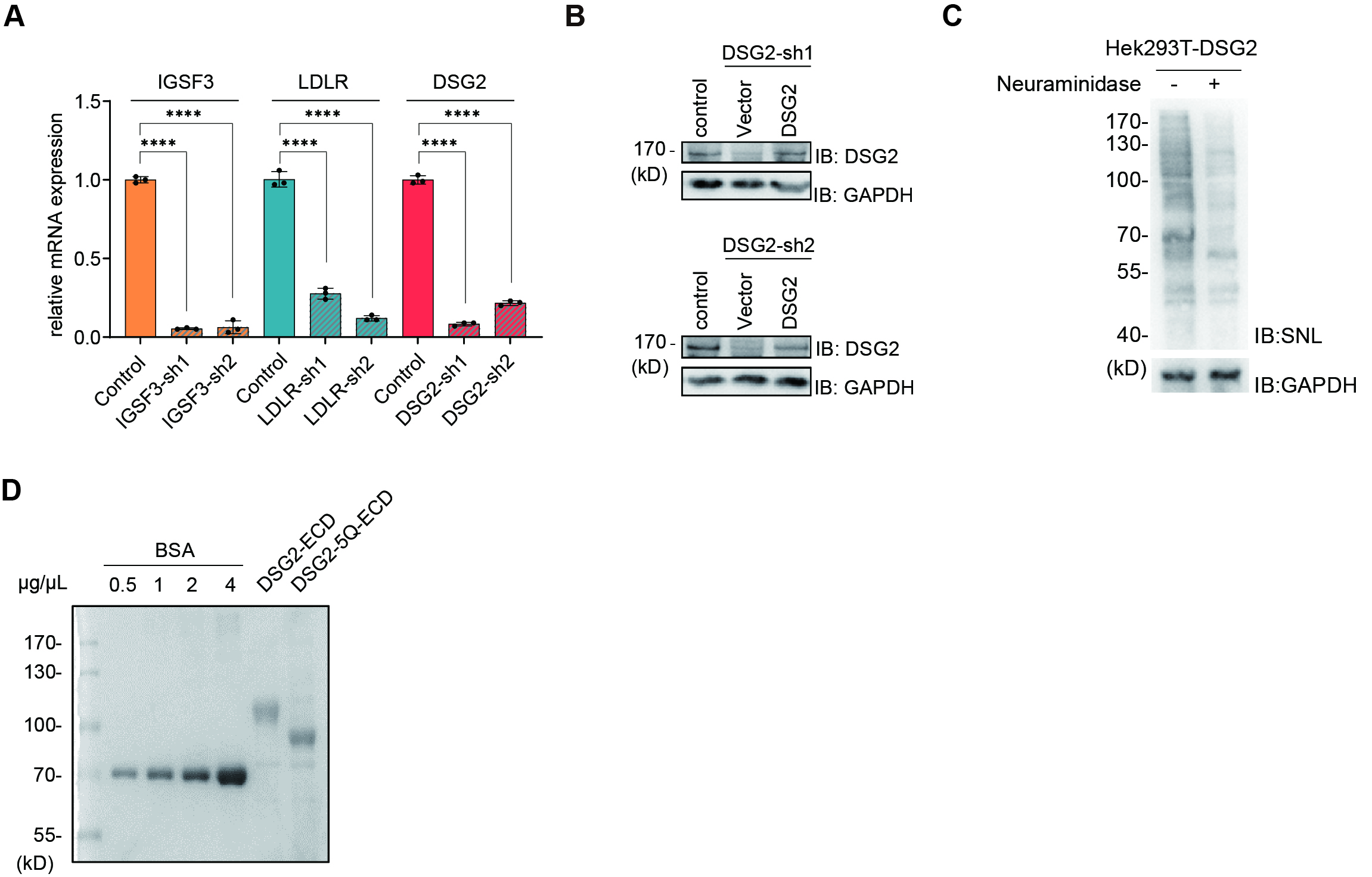
**Figure S4. A.** Confirmation of genetic knockdown of DSG2, IGSF3, and LDLR in A375 cells by RT-qPCR. A375 cells infected with the pLKO.1 empty vector were used as the control. Data are presented as mean values ± SEM (n = 3), and two-tailed *P*-values are calculated by unpaired Student's *t* test, *****P* < 0.0001. **B.** Western blot analysis of DSG2 expression in A375 cells with DSG2 knockdown by two different shRNAs and complementation with DSG2. A375 cells infected with empty vectors were used as the control. **C.** Western blot analysis of cell surface sialic acids in HEK293T cells expressing DSG2 with neuraminidase treatment using SNL. **D.** Purity confirmation and quantification of purified DSG2-ECD/5Q-ECD by reducing SDS-PAGE using coomassie stain.


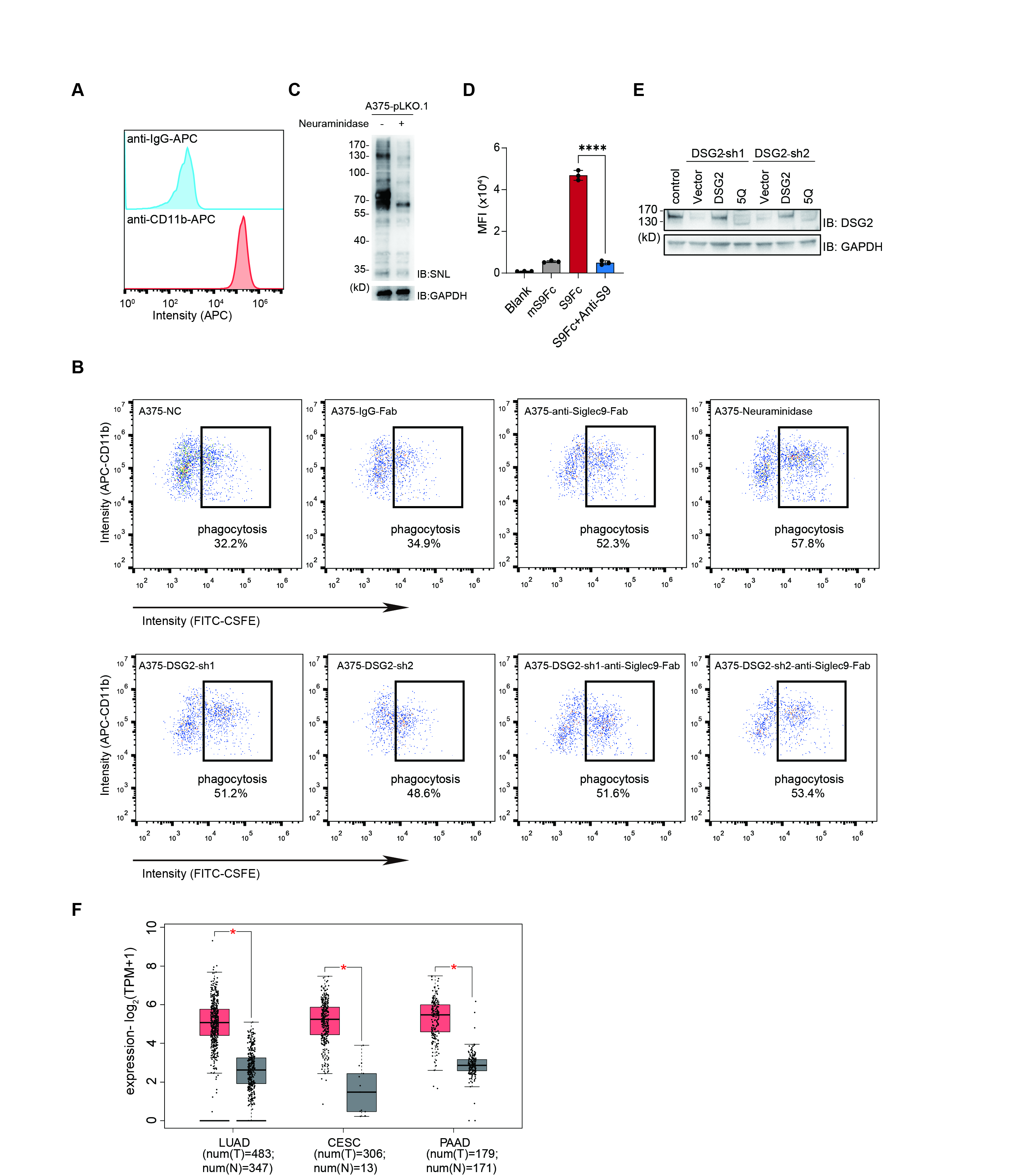


**Figure S5. A.** Flow cytometry analysis of macrophage cell surface marker CD11b expression. Macrophage cells were induced by M-CSF for 6 days and incubated with APC-conjugated anti-CD11b or APC-conjugated IgG. **B.** The representative flow cytometry scatter plots showing increases in phagocytosis of A375 by macrophages after ant-Siglec-9 Fab treatment, neuraminidase treatment, or knockdown of DSG2. Phagocytosis percentage was defined as the percentage of CD11b^+^/FITC^+^ cells among CD11b^+^ cell population. **C.** Western blot analysis of cell surface sialic acids in mock-knockdown A375 cells with neuraminidase treatment using SNL. **D.** Pre-complexing anti-Siglec-9 antibody with S9Fc can block the binding of Siglec-9 to A375 cells. **E.** Western blot analysis of DSG2 expression in A375 cells with DSG2 knockdown by two different shRNAs and complementation with DSG2-WT or DSG2-5Q. A375 cells infected with empty vectors were used as the control. **F.** RNAseq data in TCGA and GTEx database showing DSG2 overexpression in LUAD, CESC, PAAD tumor samples versus normal tissues. Sample numbers included in the analysis were shown at the bottom, and two-tailed *P*-values are calculated by unpaired Student's *t* test, **P* < 0.05.


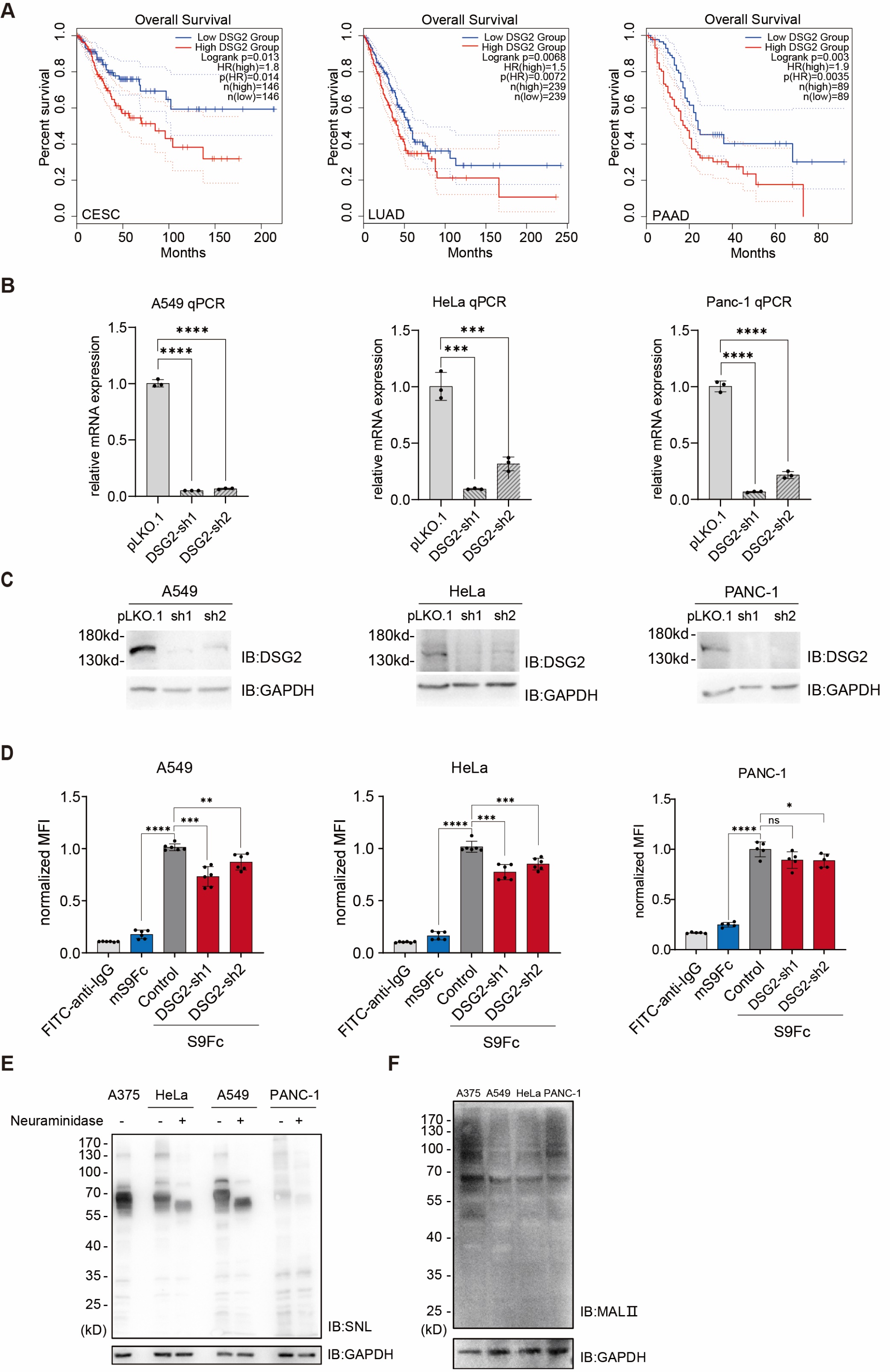


**Figure S6. A.** Overall survival analysis of patients with CESC, LUAD, and PAAD in TCGA database stratified by median DSG2 expression. HR, hazard ratio from Cox Proportional Hazards model. **B.** RT-qPCR analysis of DSG2 mRNA expression in A549, HeLa, and PANC-1 cells with DSG2 knockdown by two different shRNAs. A375 cells infected with pLKO.1 empty vector were used as the control. Data are presented as mean values ± SEM (n = 3), and two-tailed *P*-values are calculated by unpaired Student's *t* test, *****P* < 0.0001, ****P* < 0.001. **C.** Western blot analysis of DSG2 expression in A549, HeLa, and PANC-1 cells with DSG2 knockdown by two different shRNAs. A375 cells infected with pLKO.1 empty vector were used as the control. **D.** Flow cytometry analysis of Siglec-9 binding to A549, HeLa, and PANC-1 cells with DSG2 knockdown by two different shRNAs. Cells infected with pLKO.1 empty vector were used as a control. Data are presented as mean values ± SEM (n = 6 for A549 and HeLa, n = 5 for PANC-1), and two-tailed P-values are calculated by unpaired Student's *t* test, *****P* < 0.0001, ****P* < 0.001, ***P* < 0.01, **P*< 0.05. **E**. Western blot analysis of cell surface α2-3-linked sialic acids in A375, HeLa, A549, and PANC-1 cells using SNL. **F**. Western blot analysis of cell surface α2-6-linked sialic acids in A375, A549, HeLa and PANC-1 cells using MAL Ⅱ.


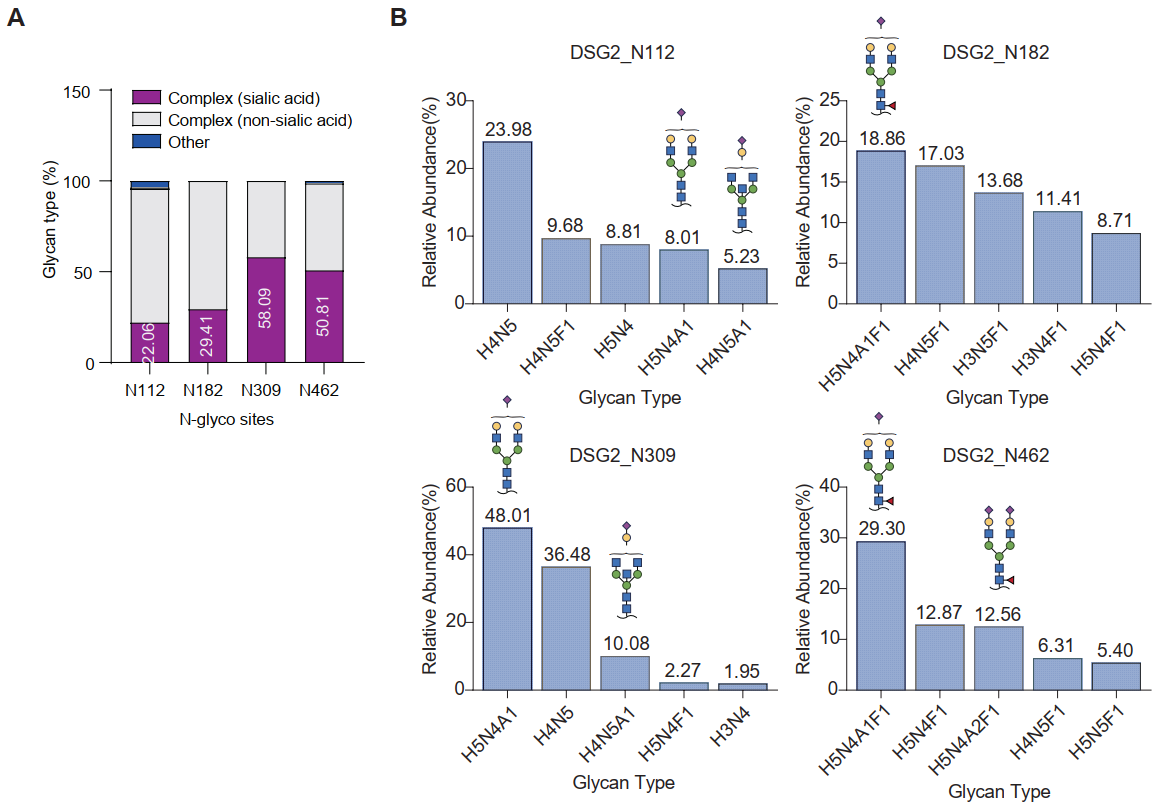


**Figure S7. A.** The analysis of sialic acid-bearing *N*-glycans of known *N*-glycosylation sites in DSG2 using Glyco-decipher. **B.** *N*-glycosylation type analysis of DSG2.
